# Cytosolic Internalization of Anti-DNA Antibodies by Human Monocytes Induces Production of Pro-inflammatory Cytokines Independently of the Tripartite Motif-Containing 21 (TRIM21)-Mediated Pathway

**DOI:** 10.3389/fimmu.2018.02019

**Published:** 2018-09-04

**Authors:** Hyunjoon Park, Minjae Kim, Youngsil Seo, Yeonkyoung Ham, Mi-Young Cho, Myung-Hee Kwon

**Affiliations:** ^1^Department of Biomedical Sciences, Graduate School, Ajou University, Yeongtong-gu, Suwon, South Korea; ^2^Department of Microbiology, Ajou University School of Medicine, Suwon, South Korea

**Keywords:** anti-DNA antibody, internalizing IgG, inflammatory cytokines, Fc-dependent signaling, human monocytes

## Abstract

Anti-DNA autoantibodies are a hallmark of systemic lupus erythematosus (SLE). A subset of anti-DNA IgG autoantibodies is cell-internalizable; thus they can enter living cells in the form of free IgG. However, the contribution made by the Fc region of internalized free-form IgG to the cytokine response has not been studied, despite the recent discovery of tripartite motif-containing 21 (TRIM21), a cytosolic Fc receptor involved in immune signaling. This study used an internalizable IgG anti-DNA antibody (3D8) to examine the cytokine responses of human monocytes to the Fc region of cytosolic free IgG. Internalization of 3D8 IgG and a 3D8 single-chain variable fragment-Fc (scFv-Fc) induced production of IL-8 and TNF-α via activation of NF-κB. By contrast, a 3D8 scFv (comprising variable domains alone) did not. This suggests Fc-dependent cytokine signaling. A 3D8 IgG-N434D mutant that is not recognized by TRIM21 induced greater production of cytokines than 3D8 IgG. Moreover the amounts of cytokines induced by 3D8 IgG in TRIM21-knockdown THP-1 cells were higher than those in control cells, indicating that cytokine signaling is not mediated by TRIM21. The results suggest the existence of a novel Fc-dependent signaling pathway that is activated upon internalization of IgG antibodies by human monocytes.

## Introduction

Antibodies can enter cells passively either by binding to constitutively internalized cell surface molecules or by forming immune complexes (ICs) with invading pathogens. Once a pathogen-antibody complex reaches the cytosol of non-immune cells, it is recognized by tripartite motif-containing 21 (TRIM21), a receptor for the antibody Fc region (1, 2). TRIM21 has Fc-dependent effector and signaling functions. The effector function, called antibody-dependent intracellular neutralization or intracellular antibody-mediated degradation, leads to rapid, VCP/p97-assisted proteasomal degradation of pathogen particles (2, 3). At the same time, TRIM21 catalyzes formation of free K63-linked ubiquitin chains, which trigger immune signaling pathways and lead to production of pro-inflammatory chemokines such as CCL5 and CXCL10 and cytokines such as TNF-α, IL-6, and IFN-β (4, 5).

Anti-DNA autoantibodies are a hallmark of an autoimmune disease called systemic lupus erythematosus (SLE); indeed, the titer of these antibodies in serum correlates with disease activity (6, 7). These antibodies form ICs with DNA and play a key role in activating signaling pathways that trigger cytokine production (8–10). For example, DNA-containing ICs bind to Fc receptors expressed by plasmacytoid dendritic cells (pDCs) and are then phagocytosed and internalized; these internalized ICs are recognized by TLR9, which then induces production of type I IFN and other pro-inflammatory cytokines (8, 9). In human monocytes, DNA-ICs induce production of IL-1β by activating the NLRP3 inflammasome (10). Free anti-DNA autoantibodies (i.e., not part of ICs) also activate cytokine signaling pathways, although the mechanisms are different (11–13). For example, production of IL-2 by Jurkat T cells is triggered via interaction with phosphoglycerate kinase 1 (11), production of IL-6 by human mesangial cells is triggered by antibody binding to annexin II followed by internalization (12), and production of IL-1β and IL-17A by monocytes/macrophages from SLE patients is triggered by interaction with TLR4, which then activates the NLRP3 inflammasome (13). Thus, in these cases cytokine signaling induced by free-form anti-DNA antibodies and by IC forms is determined by their DNA-binding properties and cross-reactivity, which are themselves determined by the distinct heavy-chain-variable (V_H_) and light-chain-variable (V_L_) regions of the antibody.

As mentioned above, a subset of anti-DNA IgG autoantibodies enters living cells in a free form Yanase et al. (14), Seddiki et al. (15), Zack et al. (16), and Song et al. (17). The mechanism of entry is distinct from that used by other antibodies, which enter cells passively by coating invading pathogens. The relationship between the cytokine signaling function of TRIM21 and SLE-like autoimmune disease activity has been examined in mouse models, but the results are contradictory (18). Thus, it is still not clear whether the Fc region of internalized IgG is recognized by TRIM21 in the cytosol, resulting in activation of TRIM21-mediated signaling pathways and culminating in production of inflammatory cytokines that exacerbate the clinical manifestations of SLE.

Here, we examined the ability of an internalizing free-form anti-DNA IgG antibody to trigger production of pro-inflammatory cytokines by human cells in an Fc-dependent manner. To do this, we used a cell-internalizable chimeric anti-DNA antibody (3D8 IgG) in which the V_H_ and V_L_ regions, which are derived from a mouse model of SLE (MRL-*lpr/lpr* mouse), were grafted onto a human IgG1 backbone. The 3D8 single-chain variable fragment (scFv) comprises only the V_H_ and V_L_ regions of the 3D8 antibody, retains DNA-binding activity, and enters cells by binding to heparan sulfate proteoglycans (HSPGs) and chondroitin sulfate proteoglycans (CSPGs) expressed on the cell surface; from there it localizes to the cytosol (19). The 3D8 scFv and 3D8 scFv-Fc antibodies were used as negative and positive controls, respectively, to verify whether the Fc region of IgG triggers cytokine responses. A 3D8 IgG-N434D mutant, which does not interact with TRIM21, was used to examine involvement of TRIM21 in cytokine responses.

Unexpectedly, we found that the Fc region of the internalizing 3D8 IgG antibody induced production of IL-8 and TNF-α in human monocytes via a pathway different from the TRIM21 pathway. These findings suggest the existence of a novel and potent intracellular Fc sensor that triggers human monocytes to produce pro-inflammatory cytokines in response to internalization of free antibody.

## Materials and methods

### Cell culture

HeLa (ATCC® number: CCL-2™) and HEK293T (ATCC® number: CRL-3216™) cells were maintained in Dulbecco Modified Eagle Medium (DMEM; Welgene Inc., Kyungsan-si, South Korea). THP-1 (ATCC® number: TIB-202) and Jurkat (ATCC® number: TIB-152) cells were maintained in RPMI 1640 medium (Welgene Inc.). DMEM and RPMI 1640 media were supplemented with 10% fetal bovine serum, 100 U/ml penicillin, and 100 μg/ml streptomycin (Welgene Inc.). All cells were cultured at 37°C/5% CO_2_. Human peripheral blood mononuclear cells (PBMCs) were isolated from healthy donor blood by density-gradient centrifugation on Ficoll-Paque (GE Healthcare, Little Chalfont, UK). Subsequently, CD14^+^ monocytes were isolated from PBMCs by magnetic-activated cell sorting using a Human CD14 Positive Selection Kit (Thermo Fisher Scientific, Waltham, MA, USA) and then cultured in RPMI 1640 medium supplemented with 10% fetal bovine serum. The study was carried out in accordance with ethical guidelines and recommendations set down by the Research Ethics Committee of Ajou University Hospital. The protocol was approved by the Ethics Committee. All subjects provided written informed consent in accordance with the Declaration of Helsinki.

### Protein preparation

FreeStyle HEK293F cells (Thermo Fisher; cat# R79007), which have been adapted to serum-free suspension culture, were used as a host for protein expression. Cells (100 ml; concentration, 1 × 10^6^ cells/ml) were seeded in a 500 ml flask (Corning, NY, USA; cat# 431145) 24 h prior to transfection to ensure that they reached the appropriate density (2 × 10^6^ cells/ml) at the time of transfection. Cells were cultured in serum-free FreeStyle 293 medium (Invitrogen, Carlsbad, CA, USA; cat# 12338) at 37°C/8% CO_2_ on an orbital shaker platform (DAIHAN Scientific, Wonju-si, South Korea [model SHD-2D]) rotating at 130 rpm. KV10 plasmids encoding wild-type (wt) 3D8 IgG, 3D8 derivatives (IgG-N434D, scFv-Fc, scFv, and IgG-G236R/L328R), and human IgG1-Fc fragment were transiently transfected into 100 ml of FreeStyle HEK293F cells using polyethylenimine (PEI) reagent (average molecular weight, 25 kDa; Polysciences, Warrington, PA, USA; cat# 23966-2). Briefly, PEI reagent (400 μg) was incubated with plasmid DNA (200 μg) at room temperature (RT) for 10 min and then inoculated onto 100 ml of cells to achieve a final PEI concentration of 4 μg/ml. After 7 days, the culture supernatant was harvested by centrifugation and clarified by filtration through a 0.45 μm cellulose acetate filter (Sartorius, Goettingen, Germany). Next, 3D8 IgG, 3D8 scFv-Fc, 3D8 IgG-G236R/L328R, and IgG1-Fc were purified by affinity chromatography on Protein A (GE Healthcare). The 3D8 IgG-N434D and 3D8 scFv antibodies were purified by affinity chromatography on a Capto L column (GE Healthcare), according to the manufacturer's guidelines. All eluted proteins were dialyzed against phosphate buffered saline (PBS, pH 7.4) and sterilized by filtration through a 0.22 μm cellulose acetate membrane filter. Polyclonal human IgGs were purchased from Sigma-Aldrich (St. Louis, MO, cat# I8640).

### Size exclusion chromatography (SEC)

SEC analyses of purified wt 3D8 IgG, 3D8 IgG N434D, and human IgG were performed using a Shimadzu UFLC system (DGU-20A3) fitted with a TSK G3000SWXL size exclusion column (7.8 × 300 mm; Toso Haas). The mobile phase was 100 mM HEPES/85 mM HNaSO_4_ (pH 6.8) and the flow rate was 1 ml/min. Proteins (1 mg/ml) were injected in PBS (30 μl). Chromatograms were obtained by monitoring absorbance at 280 nm.

### Enzyme-linked immunosorbent assay (ELISA)

To characterize cytokine profiles, cells seeded in 48-well plates (1 × 10^5^ cells per well) were treated with 5 μM 3D8 IgG for 24 or 48 h. The culture supernatants were collected and assayed for inflammatory cytokines using a Human Inflammatory Cytokines Multi-Analyte ELISArray Kit (Qiagen, Hilden, Germany; cat# MEH-004A). To measure the concentrations of IL-8 and TNF-α, THP-1 cells, and human CD14^+^ monocytes (1 × 10^5^ cells per well) in 48-well plates were treated with 3D8 proteins (5 μM) for 6 h. The concentration of cytokines in the culture supernatants was then measured using IL-8 and TNF-α ELISA kits (BioLegend, San Diego, CA, USA). If necessary, THP-1 cells were pre-treated for 1 h at 37°C with physiological inhibitors (10 μg/ml chlorpromazine (CPZ; Sigma-Aldrich), 5 mM methyl-β-cyclodextrin (MβCD; Sigma-Aldrich), 1 μg/ml cytochalasin D (Cy-D; Sigma-Aldrich), 10 μg/ml human Fc blocker (BD Biosciences, Franklin Lakes, NJ, USA), 5–10 μM polyclonal human IgG, 5 μM TLR9 inhibitor [a synthetic oligonucleotide containing the immunosuppressive motif TTAGGG (InvivoGen, San Diego, CA, USA; cat# tlrl-ttag151)], and 5 μM negative control oligonucleotide for the TLR9 inhibitor (InvivoGen; cat# tlrl-ttagc) prior to treatment with 5 μM 3D8 IgG.

Otherwise, cells were pre-treated for 1 h with DNase I (New England Biolabs, Ipswich, MA, USA), genomic DNA isolated from THP-1 cells using a Purelink™ genomic DNA mini kit (Thermo Fisher Scientific), 0.5 μM 5z-7-oxozeaenol (Sigma-Aldrich; cat# O9890), 200 nM IKK inhibitor VII (Calbiochem, Burlington, MA, USA; cat# 401486), 10 μM SB202190 (Calbiochem; cat# 559388), 50 μM PD98059 (Calbiochem; cat# 513000), or 20 μM SP600125 (Sigma-Aldrich; cat# S5567) prior to exposure to 5 μM 3D8 IgG.

To measure cytokine release by 3D8 IgG in the presence of soluble antigens (DNA or heparin), 3D8 IgG was pre-incubated for 30 min at RT with heparin (Sigma-Aldrich; cat# H3149) or genomic DNA isolated from THP-1 cells. Next, the mixture was added to THP-1 cells at a final concentration of 5 μM 3D8 IgG, 10–40 μg/ml genomic DNA, and 10 μg/ml heparin.

### Confocal microscopy

The day before use, THP-1 cells were seeded onto glass coverslips coated with poly-L-lysine, which were then placed in 24-well plates (1 × 10^5^ cells/well). Cells were incubated with 5 μM each of the 3D8 antibodies for 6 h at 37°C, fixed for 10 min at RT in 4% paraformaldehyde (PFA) in PBS, and permeabilized by incubating for 10 min at RT with Perm-buffer (1% BSA, 0.1% saponin, and 0.1% sodium azide in PBS). Cells were then incubated for 1 h at 4°C with Dylight 550-conjugated goat anti-human IgG/Fc (Abcam, Cambridge, UK; cat# 97004) to detect wt 3D8 IgG, 3D8 IgG-N434D, 3D8 scFv-Fc, and polyclonal human IgGs. To detect 3D8 scFv, cells were incubated for 1 h at 4°C with a primary antibody (polyclonal rabbit anti-3D8 scFv), followed by TRITC-conjugated goat anti-rabbit IgG (Sigma-Aldrich; cat# T6778). Each incubation step was followed by three washes with cold PBS (pH 7.2). Nuclei were stained with Hoechst 33342 (Thermo Fisher Scientific; cat# 62249) for the last 10 min of incubation at RT. Cells (on coverslips) were mounted in Vectashield anti-fade mounting medium (Vector Labs, Burlingame, CA, USA) and observed under a Zeiss LSM 710 laser confocal microscope.

### Flow cytometry

To detect binding of 3D8 IgG to the cell surface, THP-1 cells (1 × 10^6^ cells per sample) were treated for 1 h at 37°C with an FcγR blocker (final concentration, 10 μg/ml). Next, THP-1 cells were exposed to 3D8 IgG (5 μM) for 1 h at 4°C. After washing with PBS, cells were fixed for 10 min at RT with 4% PFA in PBS. Then, the cells were incubated with a primary goat anti-human kappa chain antibody (Thermo Fisher Scientific; cat# 31129), followed by a secondary FITC-conjugated rabbit anti-goat IgG antibody (Thermo Fisher Scientific; cat# 31533) (each for 1 h at 4°C). This was followed by three washes with cold PBS. Finally, cells were analyzed by flow cytometry using a FACSCanto II cytometer (BD Biosciences).

To detect binding of 3D8 IgG on the cell surface in the presence of heparin, 3D8 IgG was pre-mixed for 30 min at RT with heparin and then added to THP-1 cells (final concentrations, 5 μM 3D8 IgG and 10 μg/ml heparin) for 1 h at 4°C. After washing with PBS, cells were fixed for 10 min at RT with 4% PFA in PBS. Cells were then subjected to the procedures described above.

To examine cellular internalization of 3D8 IgG in the presence of soluble DNA, THP-1 cells were treated for 6 h at 37°C with a mixture of 3D8 IgG and genomic DNA (isolated from THP-1 cells) (final concentration, 5 μM 3D8 IgG and 10–40 μg/ml genomic DNA). Cells were washed three times with cold PBS, fixed with 4% PFA for 10 min at RT, and then permeabilized by treatment for 10 min at RT with Perm-buffer. Cells were then subjected to the procedures described above.

### Quantitative reverse transcription-PCR (qRT-PCR)

THP-1 cells seeded in 12-well plates (1 × 10^6^ cells per well) were treated with 3D8 protein (5 μM) for 6 h. Total RNA was extracted using an RNeasy Mini Kit (QIAGEN). First-strand cDNA was synthesized using a PrimeScript™ RT reagent Kit (Takara, Kusatsu, Japan; cat# RR037A), and quantitative reverse transcription-PCR (qRT-PCR) was carried out using the SYBR Premix Ex™ Taq II kit (Takara; cat# RR820A) and an ABI 7300 qPCR instrument (Applied Biosystems). The primers used for real-time RT-PCR were as follows: human IL-8, 5′-GAAGGACCTAGGACGGAAGG-3′ (forward) and 5′-GGGTGG AAAGGTTTGGAGTATG-3′ (reverse); human TNF-α, 5′-CTGCTGCACTTTGGAGTGAT-3′ (forward) and 5′-AGATGATCTGACTGCCTGGG-3′ (reverse); β-actin, 5′-CATGTACGTTGCTATCCAGGC-3′ (forward) and 5′-CTCCTTAATGTCACGCACGAT-3′ (reverse). Each sample was prepared in triplicate, and negative controls (reactions without template DNA) were run simultaneously. Relative expression of target genes was quantified by calculating the cycle threshold (CT) (2-ΔΔCt) using the average CT value for each sample.

### Lentiviral transduction

For shRNA-induced knockdown of TRIM21, THP-1 cells were transduced with lentiviral particles encoding shRNA (GCAGCACGCTTGACAATGA) specific for human TRIM21 (20). To examine activation of NF-κB, THP-1 cells were transduced with lentiviral particles carrying luciferase genes under the control of a NF-κB-responsive promoter (System Bioscience Inc., Palo Alto, CA, USA; cat# TR012PA). Transduced THP-1 cells were selected with 2 μg/ml puromycin (Gibco) to establish stable cell lines.

### Luciferase reporter assay

THP-1 cells transduced with the NF-κB reporter were seeded (3 × 10^5^ cells/well) in 24-well plates and incubated for 24 h with each 3D8 protein (5 μM). Where necessary, cells were treated for 1 h with 5z-7-oxozeaenol (0.5 μM). Cells were then assayed for firefly luciferase activity using a luciferase assay system (Promega, Madison, WI, USA; cat# E1500). Luciferase activity was expressed in terms of -fold induction relative to normalized luciferase activity in transduced THP-1 cells not treated with IgGs or drugs, which was set as 1.0.

### Western blotting

THP-1 cells were lysed in lysis buffer (20 mM Tris-HCl, 150 mM NaCl, 2 mM EDTA, and 1% Triton-X100) containing a protease inhibitor cocktail (Roche, Basel, Switzerland; cat# 04693116001). The protein concentration in cell lysates was measured using a BCA Protein assay kit (Thermo Fisher Scientific; cat# 23227). Equivalent amounts of protein (20 μg) were separated on 4–20% gradient SDS-PAGE gels (under reducing conditions) and transferred to PVDF membranes (Millipore Corp.). Transferred proteins were subjected to immunoblotting with antibodies specific for TRIM21 (Santa Cruz Biotechnology, Dallas, TX, USA; cat# sc-25351), IgG-Fc (Abcam; cat# ab97221), Ig kappa chain (Abcam; cat# 134083), β-actin (Bethyl Laboratories, Montgomery, TX, USA; cat# A300-491A), or GAPDH (Santa Cruz Biotechnology; cat# sc-32233), followed by detection with HRP-conjugated horse anti-mouse IgG (Cell Signaling Technology, Danvers, MA, USA; cat# 7076) or HRP-conjugated anti-rabbit IgG (Thermo Fisher Scientific; cat# 81-6120). Signals were detected using an ECL kit (GE Healthcare; cat# RPN2106).

### Immunoprecipitation (IP)

Cells were seeded in 6-well plates (1 × 10^7^ cells/well) and then treated with wt 3D8 IgG and 3D8 IgG-N434D (each at 5 μM). At 6 h post-treatment, cell lysates were prepared using an IP kit (Thermo Fisher Scientific; cat# 26148), according to the manufacturer's instructions. Briefly, after lysis with ice-cold IP Lysis buffer containing a protease inhibitor cocktail, 500 μg of cell lysate was subjected to immunoprecipitation (IP) with Protein A/G coupled to a resin. After washing the resin, immunoprecipitated proteins were eluted and resolved on 4–20% gradient SDS-PAGE gels, followed by immunoblotting with antibodies specific for TRIM21 (Santa Cruz Biotechnology; cat# sc-25351), IgG-Fc, or Ig kappa chain (both from Abcam; cat# 134083).

## Results

### Treatment of THP-1 cells with 3D8 induces secretion of IL-8 and TNF-α

**Figure 1:**
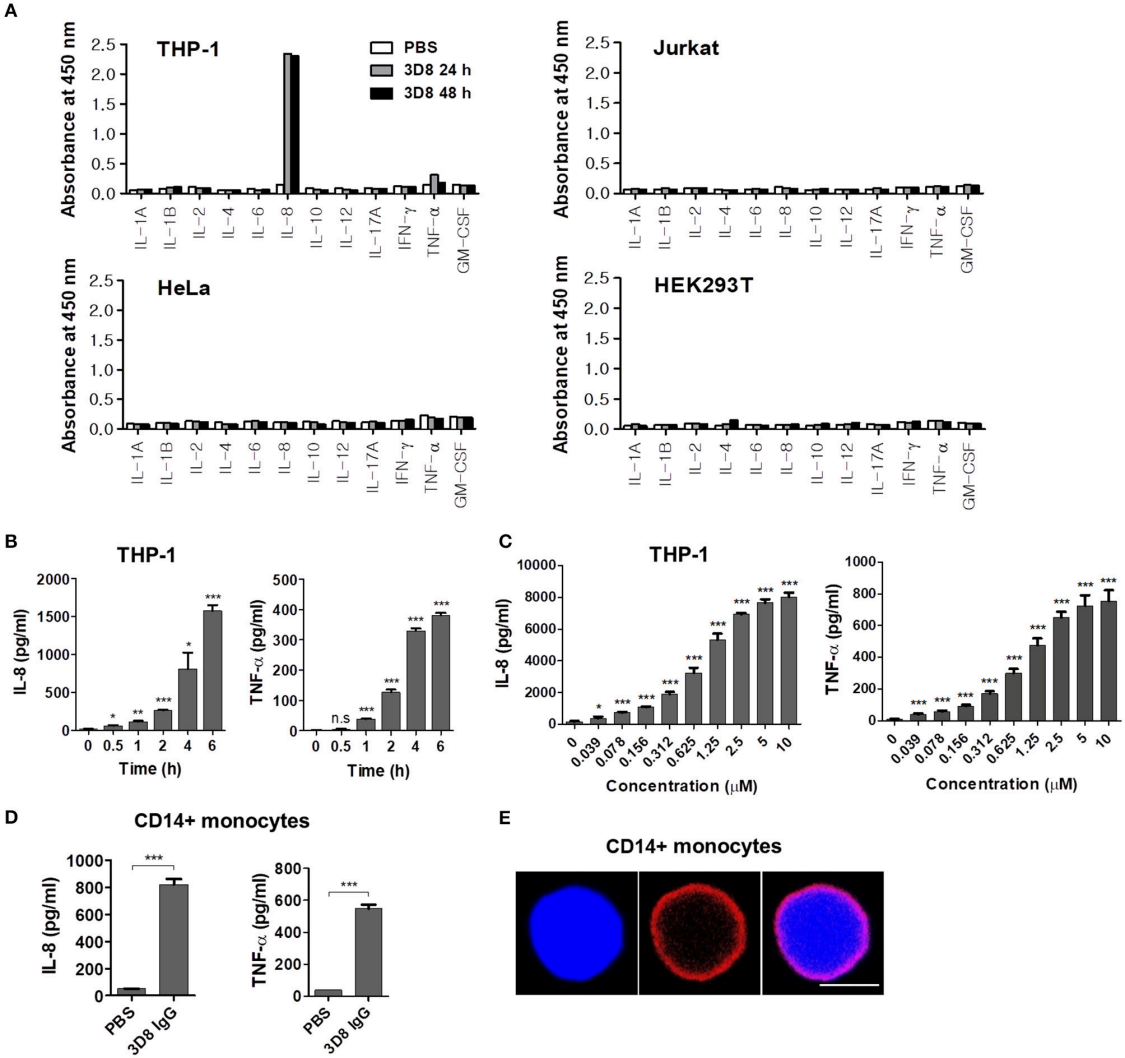
Cytokine secretion by human cells treated with internalizable 3D8 IgG antibodies. **(A)** Cytokine production by different cell lines. Cells were exposed to 10 μM 3D8 IgG for 24 or 48 h, and cytokine secretion was analyzed (in duplicate) using ELISA kits. **(B,C)** THP-1 cells were treated with 5 μM 3D8 IgG antibodies for the indicated times at 37°C **(B)** or with different concentrations of 3D8 antibody for 6 h at 37°C **(C)**. The amount of IL-8 and TNF-α in culture supernatants was analyzed using ELISA kits. **(D)** Cytokine secretion by human primary CD14^+^ monocytes exposed to 5 μM 3D8 IgG antibodies. **(E)** Confocal microscopy. Human primary CD14^+^ monocytes were exposed to 5 μM 3D8 IgG antibodies for 6 h at 37°C. After fixation and permeabilization, cells were incubated with Dylight 550-conjugated goat anti-human IgG/Fc. Scale bar, 5 μm. Data are expressed as the mean ± standard error of the mean (three independent experiments). All *p*-values were calculated using a two-tailed Student's *t*-test (n.s., not significant; *p* > 0.05; *p < 0.05; **p < 0.01; and ***p < 0.001 vs. negative control).

The internalizable chimeric 3D8 IgG antibody harboring variable regions derived from a mouse anti-DNA antibody and constant regions derived from human IgG1 was prepared from a FreeStyle HEK293F cell culture. Next, two human immune cell lines, THP-1 (a monocytic leukemia cell line) and Jurkat (a T lymphoblastoid cell line), and two human non-immune cell lines, HeLa (a cervical carcinoma cell line) and HEK293T (an embryonic kidney cell line), were treated with 3D8 IgG for 24 or 48 h, and the cytokine profiles were screened by ELISA. We observed marked production of IL-8 and TNF-α by 3D8 IgG-treated THP-1 cells; this was not the case for the other cell lines tested (Figure 1A). Production of IL-8 and TNF-α by 3D8-treated THP-1 cells was both time- (up to 6 h) (Figure 1B) and dose- (up to 10 μM) dependent (Figure 1C). Next, we examined cytokine production by primary human CD14^+^ monocytes. Cells treated with 5 μM 3D8 secreted both IL-8 and TNF-α (Figure 1D). Confocal microscopy showed that internalizable 3D8 IgG localized in the cytosol (Figure 1E), suggesting that internalization of free 3D8 IgG by CD14^+^ human monocytes triggers production of IL-8 and TNF-α.

### The Fc region of internalized 3D8 IgG triggers secretion of IL-8 and TNF-α by THP-1 cells

**Figure 2:**
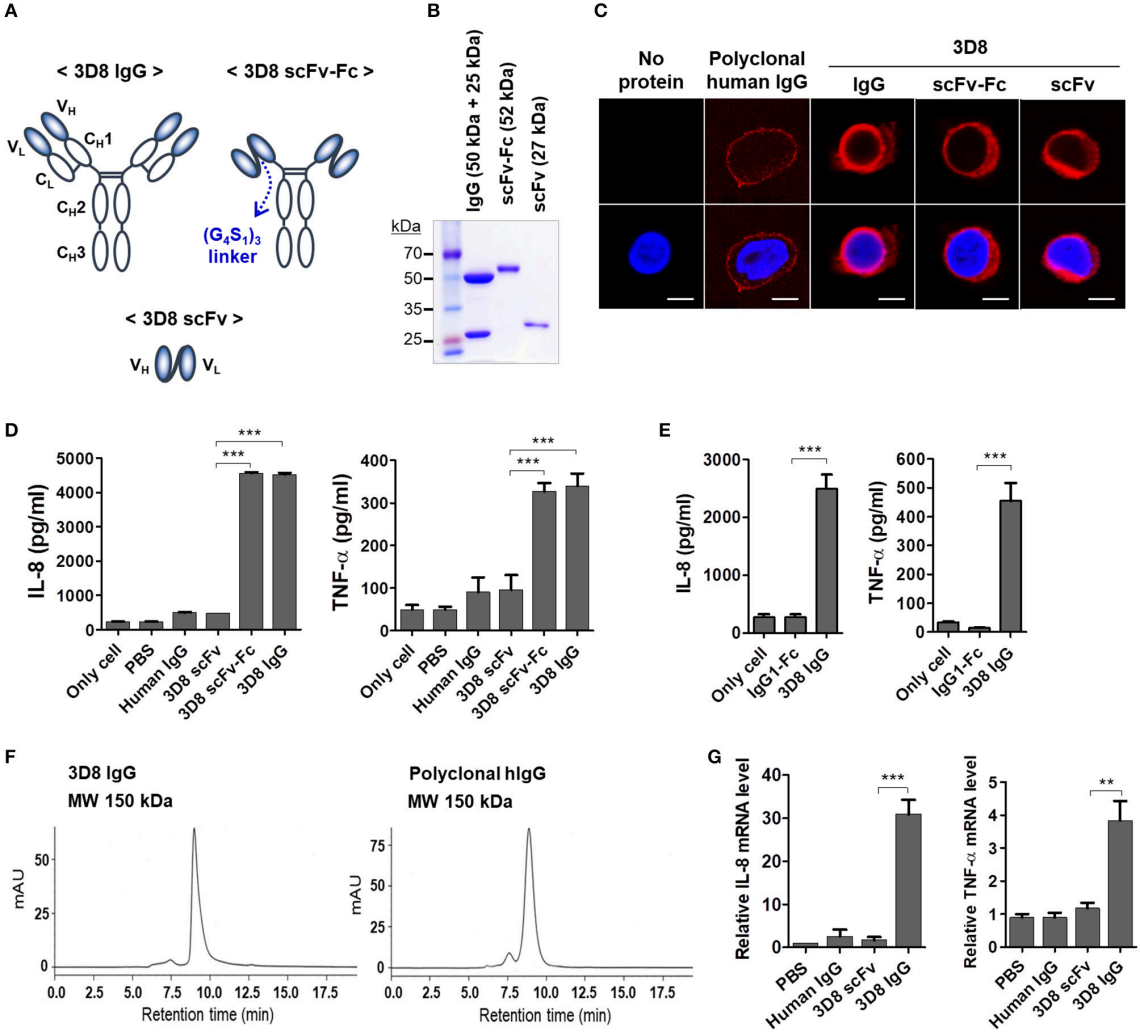
The Fc region of internalizable 3D8 IgG antibody triggers production of IL-8 and TNF-α by THP-1 cells. **(A)** Schematic representation of the different 3D8 molecules. **(B)** SDS-PAGE analysis of 3D8 antibodies purified from the culture supernatant of HEK293F cells transfected with genes encoding the different antibody formats. **(C)** Confocal microscopic analysis of intracellular localization of 3D8 antibodies. Cells were exposed to 5 μM antibodies for 6 h at 37°C. After fixation and permeabilization, cells were incubated with Dylight 550-conjugated goat anti-human IgG/Fc to detect IgG and scFv-Fc. 3D8 scFv was detected by a polyclonal rabbit anti-3D8 scFv, followed by TRITC-conjugated goat anti-rabbit IgG. **(D,E)** Cytokine secretion. Cells were exposed to 5 μM 3D8 antibodies, polyclonal human IgGs **(D)**, and 5 μM isotype control (human IgG1-Fc fragment) **(E)** for 6 h at 37°C. The amount of IL-8 and TNF-α in the culture supernatant was measured using ELISA kits. **(F)** Size exclusion chromatography of 3D8 IgG and polyclonal human IgG. The indicated molecular weights were interpolated using a standard curve set up using proteins with known mass and retention time. **(G)** Quantitative RT-PCR of mRNA encoding IL-8 and TNF-α in cells exposed to 5 μM 3D8 antibodies for 6 h at 37°C. **(D,E,G)** Data are expressed as the mean ± standard error (three independent experiments). All *p*-values were calculated using a two-tailed Student's *t*-test (**p < 0.01 and ***p < 0.001).

To determine whether the Fc region of internalized 3D8 IgG was responsible for cytokine production by THP-1 cells, we prepared two different 3D8 antibody constructs: a 3D8 scFv comprising variable regions of heavy (V_H_) and light (V_L_) chains and a 3D8 scFv-human Fc fusion protein (Figure 2A). As expected, single protein bands were detected at 27 kDa (scFv) and at 52 kDa (scFv-Fc) on a reducing SDS-PAGE gel; by contrast, two bands (one at 50 kDa and one at 25 kDa) were detected for the complete 3D8 IgG molecule (Figure 2B). Confocal microscopy revealed that all internalizable 3D8 antibodies localized to the cytosol of THP-1 cells (Figure 2C). Previous studies report internalization of 3D8 scFv into the cytosol of human epithelial (HeLa) cells, human embryonic kidney (HEK293T) cells (19, 21), and mouse dendritic (DC2.4) cells (22). By contrast, a negative control polyclonal human IgG localized to the cell surface [probably due to capture by Fcγ receptors (FcγRs)]. THP-1 cells exposed to 5 μM 3D8 IgG or 3D8 scFv-Fc for 6 h secreted marked amounts of IL-8 and TNF-α; this was not the case for cells exposed to 3D8 scFv lacking the Fc region, or for cells exposed to polyclonal human IgG (Figure 2D). Moreover, only background levels of IL-8 and TNF-α secretion were detected in THP-1 cells treated with a isotype control (human IgG1-Fc fragment) expressed and purified using the same protocol used for 3D8 IgG (Figure 2E). Size exclusion chromatography confirmed correct assembly of the purified 3D8 IgG, with no aggregation (left panel of Figure 2F), similar to the commercial polyclonal IgG (right panel of Figure 2F). Therefore, we could exclude the possibility of an accidental cytokine response triggered by protein aggregation during protein preparation. Cytokine expression correlated with that of cytokine-encoding mRNAs (Figure 2G). These results indicate that production of IL-8 and TNF-α by THP-1 cells is triggered by the Fc region of internalized 3D8 and not by the variable regions.

### Production of inflammatory cytokines triggered by internalized IgG

**Figure 3:**
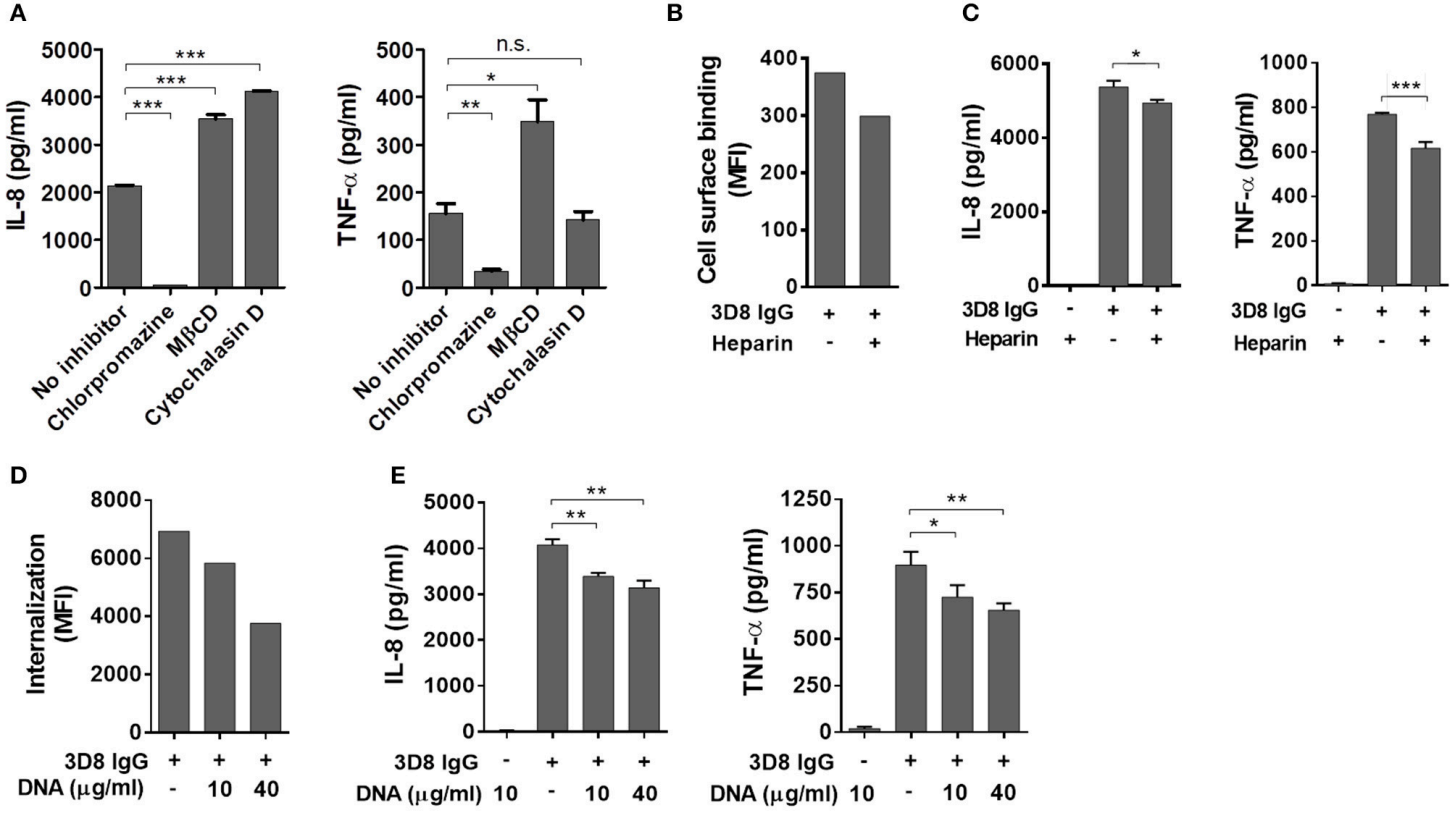
Induction of inflammatory cytokines by internalized IgG. **(A)** ELISA. Prior to exposure to 5 μM 3D8 IgG for 6 h, THP-1 cells were treated with endocytosis inhibitors [10 μg/ml chlorpromazine, 5 mM MβCD, or 1 μg/ml cytochalasin **(D)**]. The amount of IL-8 and TNF-α in the culture supernatant was measured using ELISA kits. **(B)** Binding of 3D8 IgG (5 μM) to the cell surface in the presence of 10 μg/ml heparin was examined by flow cytometry. **(C,E)** ELISA. THP-1 cells were treated with a mixture of 3D8 IgG (5 μM) and heparin (10 μg/ml) **(C)**, or with a mixture of 3D8 IgG (5 μM) and the indicated concentrations of DNA **(E)**. The amounts of IL-8 and TNF-α in the culture supernatant were measured using ELISA kits. All *p*-values were calculated using a two-tailed Student's t test (n.s., not significant; *p* > 0.05; *p < 0.05; **p < 0.01; and ***p < 0.001, vs. negative control). n.s., not significant. **(D)** Internalization of 5 μM 3D8 IgG into THP-1 cells in the presence of the indicated concentrations of DNA isolated from THP-1 cells was examined by flow cytometry.

Because internalization of IgG is prerequisite for production of inflammatory cytokines, we next investigated whether cytokine production is inhibited by treatment with pharmacological inhibitors that interfere with individual endocytic pathways. The inhibitors were CPZ (which inhibits clathrin-dependent endocytosis), MβCD (which inhibits caveolae/lipid raft endocytosis), and Cy-D (which inhibits macropinocytosis) (23). THP-1 cells were pre-treated with these inhibitors for 30 min at 37°C prior to addition of 3D8 IgG (5 μM). Cells pre-incubated with CPZ did not secrete IL-8 or TNF-α, whereas cells exposed to MβCD or Cy-D did (Figure 3A), suggesting that 3D8 IgG internalizes via clathrin-dependent endocytosis.

Next, we asked whether cytokine production by 3D8 IgG is inhibited when the antigen-binding sites are pre-occupied by heparin (a soluble analog of heparin sulfate) (24) as well as by DNA antigen. 3D8 scFv cross-reacts with negatively charged sugar chains on HSPGs and CSPGs prior to entering the cell (19). We found that binding of 3D8 IgG to the THP-1 cell surface fell by ~20% after pre-incubation with soluble heparin (Figure 3B); in addition, secretion of IL-8 and TNF-α fell by ~8 and 20%, respectively (Figure 3C). Moreover, we investigated the effect of DNA on endocytosis of 3D8 IgG and subsequent cytokine production. Internalization of 3D8 IgG into THP-1 cells fell by ~16 and 46% after pre-incubation with 10 and 40 μg/ml DNA, respectively (Figure 3D). This is because DNA interferes with the interaction between 3D8 IgG and cell surface HSPGs/CSPGs, as does soluble heparin. When 3D8 IgG internalization was reduced by ~46%, secretion of IL-8 and TNF-α was reduced by ~17 and 23%, respectively (Figure 3E). These results suggest that internalized IgG induces production of IL-8 and TNF-α by THP-1 cells.

### Production of inflammatory cytokines triggered by internalized IgG does not occur via cell surface FcγR- or intracellular TLR9-mediated signaling pathways

**Figure 4:**
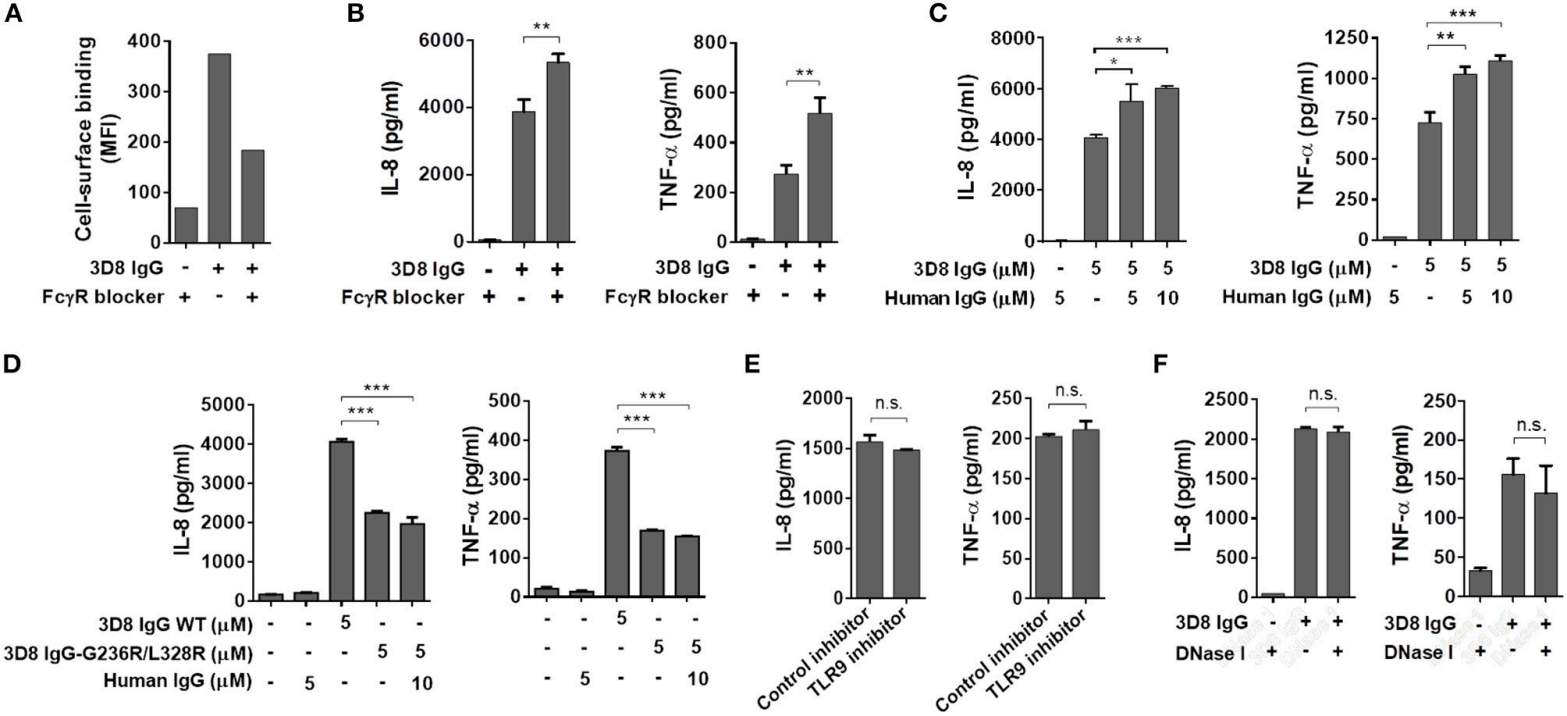
Production of inflammatory cytokines triggered by internalized IgG does not occur via cell surface FcγR- or intracellular TLR9-mediated signaling pathways. **(A)** Flow cytometry analysis of 3D8 IgG (5 μM) binding to THP-1 cells in the presence of an FcγR blocker (10 μg/ml). **(B,C,E,F)** ELISA. Prior to exposure to 3D8 IgG (5 μM) for 6 h, THP-1 cells were treated with an FcγR blocker (10 μg/ml) **(B)**, the indicated concentrations of human polyclonal IgG **(C)**, a TLR9 inhibitor (5 μM) **(E)**, or 1 U/ml DNase I **(F)** for 1 h at 37°C. **(D)** THP-1 cells were treated for 6 h at 37°C with either 3D8 IgG-G236R/L328R (5 μM) or a mixture of 3D8 IgG-G236R/L328R (5 μM) and polyclonal human IgG (10 μM). The amounts of IL-8 and TNF-α in the culture supernatant were measured using ELISA kits. All *p*-values were calculated using a two-tailed Student's *t*-test (n.s., not significant; *p* > 0.05; *p < 0.05; **p < 0.01; and ***p < 0.001).

In addition to our observation that polyclonal human IgGs do not enter cells or induce a cytokine response, even upon binding to the THP-1 cell surface via FcγRs (Figure 2), we asked whether cytokine production induced by 3D8 IgG is involved in signaling via FcγRs. THP-1 cells were pre-incubated with a commercial human FcγR blocker (10 μg/ml) or with polyclonal human IgG (5 and 10 μM) to prevent interaction between 3D8 IgG and FcγRs on the cell surface; cells were then exposed to 3D8 IgG (5 μM). Whereas binding of 3D8 IgG to the cell surface fell by 49% in the presence of the FcγR blocker (Figure 4A), secretion of IL-8 and TNF-α increased by ~37 and 89%, respectively (Figure 4B). Similarly, secretion of IL-8 and TNF-α by THP-1 cells pre-treated with polyclonal human IgG was significantly higher than that by untreated control cells (Figure 4C). The increased cytokine responses may be due to increased endocytosis of 3D8 IgG by cells pre-treated with the FcγR blocker or polyclonal IgG, both of which interfere with capture of 3D8 IgG by FcγRs. These results suggest that production of IL-8 and TNF-α is not mediated by the FcγR signaling pathway.

Treatment of cells with a 3D8 IgG mutant (3D8 IgG-G236R/L328R), the Fc region of which does not interact with FcγRs (25), led to secretion of IL-8 and TNF-α, even at low levels (~50% of the cytokine levels induced by wt 3D8 IgG). Co-treatment with mutant 3D8 IgG-G236R/L328R (5 μM) and polyclonal human IgG (10 μM) did not increase cytokine secretion compared with treatment with 3D8 IgG-G236R/L328R alone (Figure 4D). These results suggest that FcγR signaling plays no role in cytokine secretion, even if the interaction between the IgG-Fc region and an unknown intracellular Fc sensor may be affected by the mutation of Fc (G236R/L328R).

Anti-DNA antibodies bind to endogenous DNA released from dead cells to form antibody-DNA-ICs (26), which are then phagocytosed by pDCs via FcγRs prior to recognition by intracellular TLR9; this triggers production of type I IFN and pro-inflammatory cytokines, which drive pathogenesis of SLE (8, 9, 27, 28). Thus, we next examined the possibility that cytokine production occurs via TLR9 signaling stimulated by ICs comprising 3D8 IgG and DNA. Pre-treatment of THP-1 cells with a TLR9 inhibitor did not affect production of IL-8 or TNF-α upon exposure to 3D8 IgG (Figure 4E). Cells treated with DNase I prior to incubation with 3D8 IgG showed no significant change in the amount of IL-8 and TNF-α (Figure 4F). These results suggest that production of IL-8 and TNF-α triggered by internalizable IgG is not induced via a TLR9-mediated pathway.

### Production of IL-8 and TNF-α by internalizable IgG is not mediated by the TRIM21 signaling pathway

**Figure 5:**
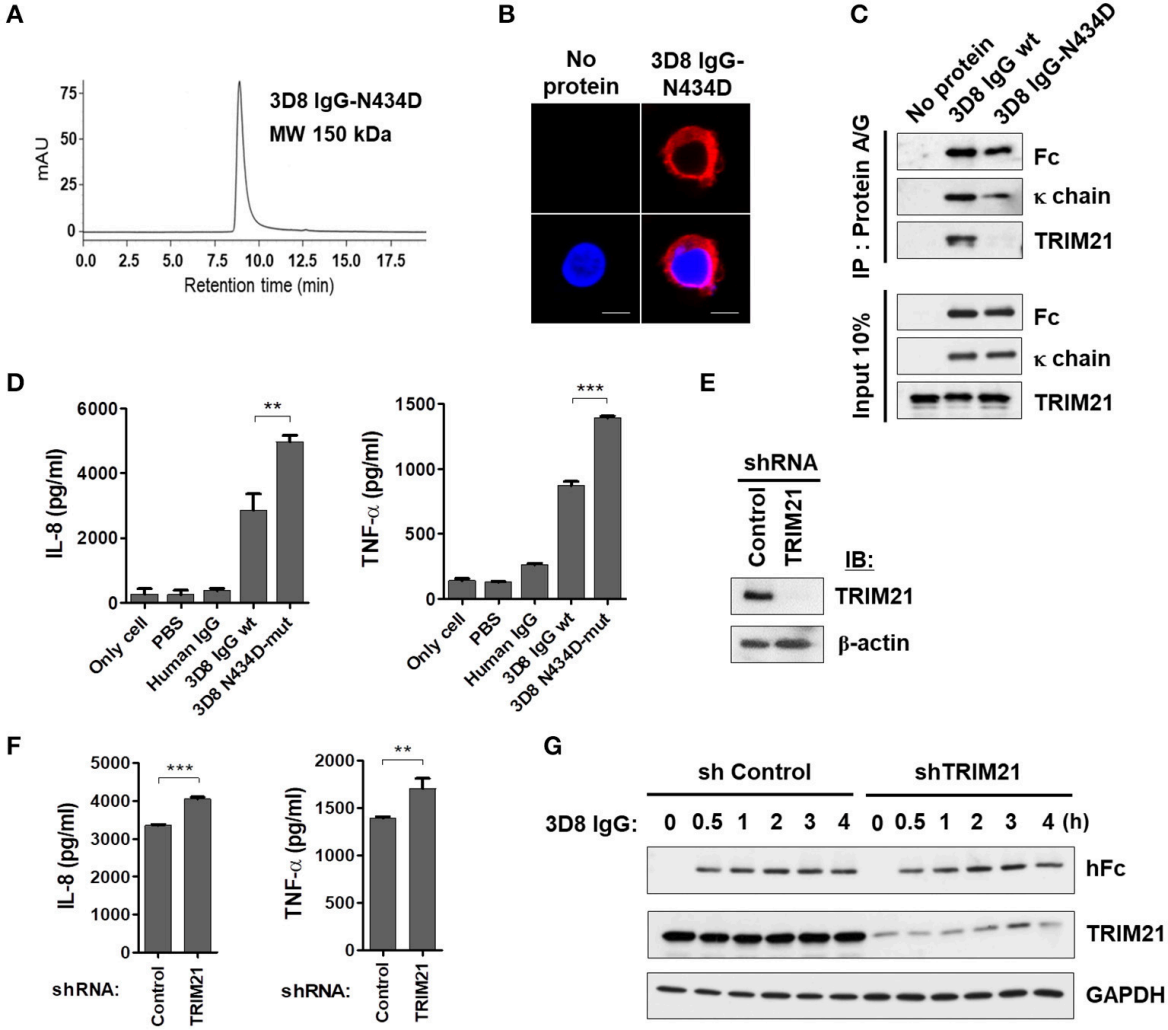
Internalizable IgG-mediated production of IL-8 and TNF-α is not mediated by TRIM21. **(A)** Size exclusion chromatography of 3D8 IgG-N434D. **(B)** Confocal microscopy. THP-1 cells were exposed to the 3D8 IgG-N434D mutant (5 μM) for 6 h at 37°C. After fixation and permeabilization, cells were stained with Dylight 550-conjugated goat anti-human IgG/Fc. **(C)** Co-immunoprecipitation of 3D8 IgG antibodies and TRIM21. THP-1 cells were exposed to 3D8 IgG antibodies (5 μM) for 6 h at 37°C. Cell lysates were then co-immunoprecipitated with Protein A/G. Samples were then analyzed by immunoblotting with antibodies specific for TRIM21, the Fc portion of human IgG, and human IgG κ light chain. Input controls comprised 10% total cell lysate. **(D)** ELISA. THP-1 cells were treated with 3D8 IgG antibodies (5 μM) for 6 h at 37°C, and the amounts of IL-8 and TNF-α in the culture supernatants were measured using ELISA kits. **(E)** Western blot analysis of shRNA-mediated knockdown of TRIM21 in THP-1 cells. **(F)** ELISA. IL-8 and TNF-α were measured in TRIM21-knockdown THP-1 and control cells exposed to 5 μM 3D8 IgG antibodies for 6 h at 37°C. **(D,F)** Data are expressed as the mean ± standard error (three independent experiments). All *p*-values were calculated using a two-tailed Student's *t*-test. Statistical significance is indicated on the graphs (**p < 0.01 and ***p < 0.001). **(G)** Immunoblot analysis of TRIM21 and human IgG H chain expression in TRIM21-knockdown THP-1 cells and control cells. GAPDH was used as a loading control.

Next, we asked whether cytokine production triggered by internalized free 3D8 IgG is mediated by TRIM21, a cytosolic Fc receptor with E3 ubiquitin ligase activity (1, 29). For this, we used a 3D8 IgG-N434D mutant that cannot interact with TRIM21 due to a single amino acid substitution in the C_H_3 domain (1, 30) as a negative control. Size exclusion chromatography revealed correct assembly of the purified 3D8 IgG-N434D, with no aggregation (Figure 5A), and internalization of 3D8 IgG-N434D into THP-1 cells was confirmed by confocal microscopy (Figure 5B). Physical interaction between 3D8 IgG and TRIM21 was confirmed in a pull-down assay in which lysates of THP-1 cells treated with 3D8 IgG proteins were precipitated with Protein A/G. We found that wt 3D8 IgG pulled down TRIM21, whereas the 3D8 IgG-N434D mutant did not, indicating an interaction between wt 3D8 IgG and endogenous TRIM21 (Figure 5C). When THP-1 cells were treated with 5 μM 3D8 IgG protein for 6 h, secretion of IL-8 and TNF-α by cells treated with the 3D8 IgG-N434D mutant was ~40% higher than that by cells treated with wt 3D8 IgG (Figure 5D). Moreover, increased production (~20%) of IL-8 and TNF-α by wt 3D8 IgG was observed in TRIM21-knockdown THP-1 cells (Figures 5E,F). These data suggest that TRIM21 does not play a key role in cytokine signaling induced by freely internalized IgG, although TRIM21 may function as a weak negative regulator of an as-yet-unknown Fc-mediated cytokine signaling pathway.

Because TRIM21 is an E3 ubiquitin ligase, we asked whether reduced cytokine production triggered by wt 3D8 IgG was related to proteasomal degradation of wt 3D8 IgG. Stable TRIM21-knockdown THP-1 cells and control THP-1 cells were incubated with wt 3D8 IgG for the indicated times, and proteins in cell lysates were detected using an anti-human IgG-Fc and anti-TRIM21 antibodies. The H chain of 3D8 IgG appeared to be neither ubiquitinated nor degraded at any time in TRIM21-knockdown THP-1 cells and control cells, indicating that TRIM21 does not degrade freely internalized IgG (Figure 5G).

### IL-8 and TNF-α are induced by internalizable IgG via activation of NF-κB through the mitogen-activated protein kinase (MAPK) signaling pathway

**Figure 6:**
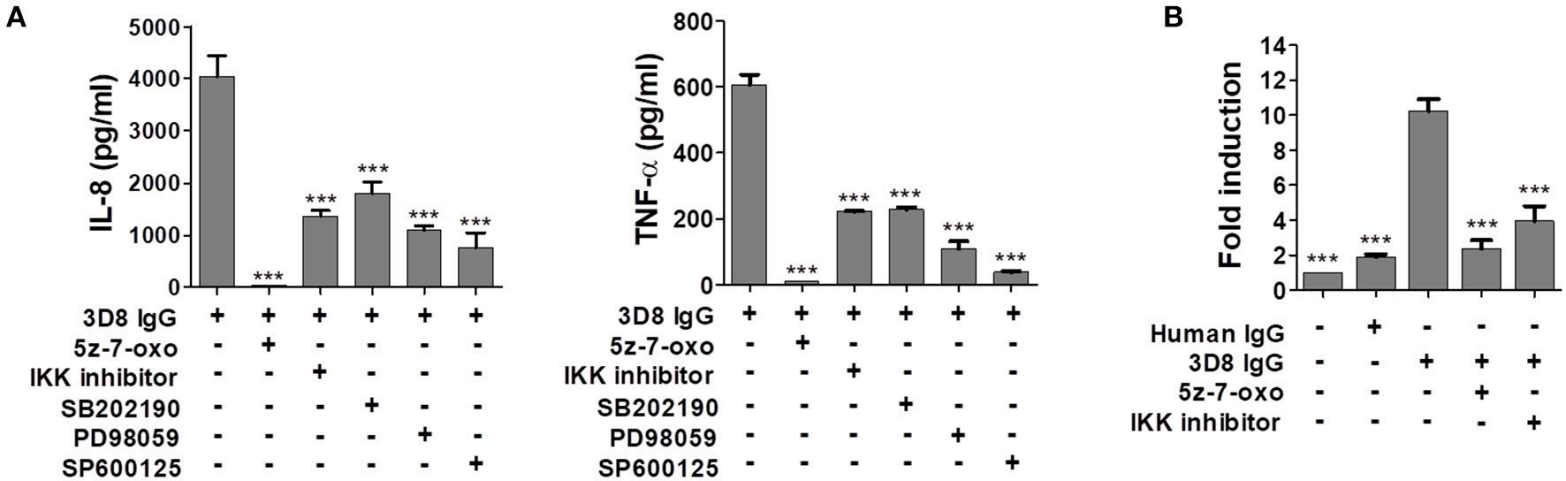
Internalizable IgG-mediated production of IL-8 and TNF-α occurs via NF-κB activation through the MAPK signaling pathway. **(A)** Pharmacological inhibition of cytokine production by THP-1 cells exposed to 3D8 IgG. Cells were pre-treated for 1 h with 0.5 μM 5z-7-oxozeaenol, 200 nM IKK inhibitor VII, 10 μM SB202190, 50 μM PD98059, or 20 μM SP600125 prior to exposure to 5 μM 3D8 IgG. The amount of IL-8 and TNF-α in the culture supernatant was measured using ELISA kits. **(B)** Luciferase assay to detect activation of the NF-κB promoter. THP-1 NF-κB reporter cells, which were established using a lentiviral system, were pre-treated for 1 h with 0.5 μM 5z-7-oxozeaenol and then incubated with 5 μM 3D8 IgG for 24 h. Cells were harvested, and luciferase assays were performed. All p values were calculated using a two-tailed Student's *t*-test (***p < 0.001, vs. treatment with 3D8 IgG alone).

Studies show that expression of the gene encoding IL-8 is regulated by NF-κB and AP-1 transcription factors via the MAPK family of proteins, which includes JNK, p38, and ERK (31, 32). Thus, we used specific inhibitors of MAPK signaling to determine whether the MAPK pathway and the TAK1-linked canonical NF-κB signaling pathway (33) are activated by 3D8 IgG. The inhibitors were 5z-7-oxozeaenol (a TAK1 inhibitor), IKK inhibitor VII (an IKKα/β inhibitor), SB202190 (a p38 inhibitor), PD98059 (an ERK inhibitor), and SP600125 (a JNK inhibitor). THP-1 cells pre-treated with all of these inhibitors showed markedly reduced production of IL-8 and TNF-α after exposure to 3D8 IgG. Compared with production after exposure to 3D8 IgG alone, IL-8 production fell by approximately 99% after exposure to antibody plus 5z-7-oxozeaenol, by 66% after exposure to antibody plus the IKK inhibitor, by 53% after exposure to antibody plus SB202190, by 73% after exposure to antibody plus PD98059, and by 81% after exposure to antibody plus SP600125. Similarly, production of TNF-α fell by approximately 92% after exposure to antibody plus 5z-7-oxozeaenol, by 63% after exposure to antibody plus IKK inhibitor, by 62% after exposure to antibody plus SB202190, by 82% after exposure to antibody plus PD98059, and by 94% after exposure to antibody plus SP600125 (Figure 6A). Taken together, these results indicate that activation of NF-κB signaling via the MAPK pathways plays a role in IL-8 and TNF-α production induced by 3D8 IgG. The role of NF-κB was confirmed by the observation that pre-treatment of cells with 5z-7-oxozeaenol or the IKK inhibitor reduced NF-κB luciferase reporter activity by 70–80% (Figure 6B).

## Discussion

Here, we show that internalization of an IgG antibody into the cytosol of human monocytes activates cytokine signaling pathways, leading to secretion of pro-inflammatory cytokines. Overproduction of cytokines plays a key role in the onset or progression of SLE (1), a chronic autoimmune inflammatory disease involving multiple organs (34). Anti-DNA autoantibodies are a hallmark of SLE, with titers correlating with disease activity (6, 7); however, it is not clear how these anti-DNA antibodies exert their pathogenic effects. Most studies examining the pathological mechanisms mediated by IgG anti-DNA antibodies addressed the properties endowed by the variable regions, including DNA-binding activity for IC formation, nuclear-transport activity to induce apoptosis, and cross-reactivity with other self-antigens (35–37). Here, we showed that two 3D8 antibody formats (3D8 IgG and 3D8 scFv-Fc) induced production of inflammatory cytokines, whereas 3D8 scFv did not, despite cytosolic localization after internalization. To the best of our knowledge, this is the first report to show that internalizing anti-DNA antibody (IgG) induced production of cytokines via the Fc region when exposed to the cytosolic compartment.

Evidence suggests that a subset of anti-DNA antibodies derived from mouse models of SLE enter a variety of living cells (14–17, 38–43); several molecules, including calreticulin, myosin 1, and equilibrative nucleoside salvage transporter (ENT) act as cell surface receptors for internalization of some anti-DNA antibodies, although there is little direct evidence that they are endocytic receptors. For example, calreticulin (15) and myosin 1 (14) were immuno-captured from cells treated with the monoclonal anti-DNA antibodies F14.6/H9.3 and H7, respectively. Also, the anti-DNA monoclonal antibody 3E10 cannot penetrate ENT-deficient cells (43). The 9D7 anti-dsDNA monoclonal antibody does not require a cell membrane receptor, even though it interacts initially with cell surface HSPGs prior to internalization (17). To date, autoreactive human anti-DNA mAbs have been isolated from SLE patients, but their internalizing ability has not been studied in detail (44–46). Moreover, the ratio of internalizing antibody to total anti-DNA antibody present in the serum of SLE patients has not been determined experimentally because no quantitative assay is available. Thus, establishing a correlation between the detected levels of internalizing antibodies in serum and the levels of inflammatory cytokines in serum may help to elucidate the role of internalizing anti-DNA antibody in the pathogenesis of SLE.

**Figure 7:**
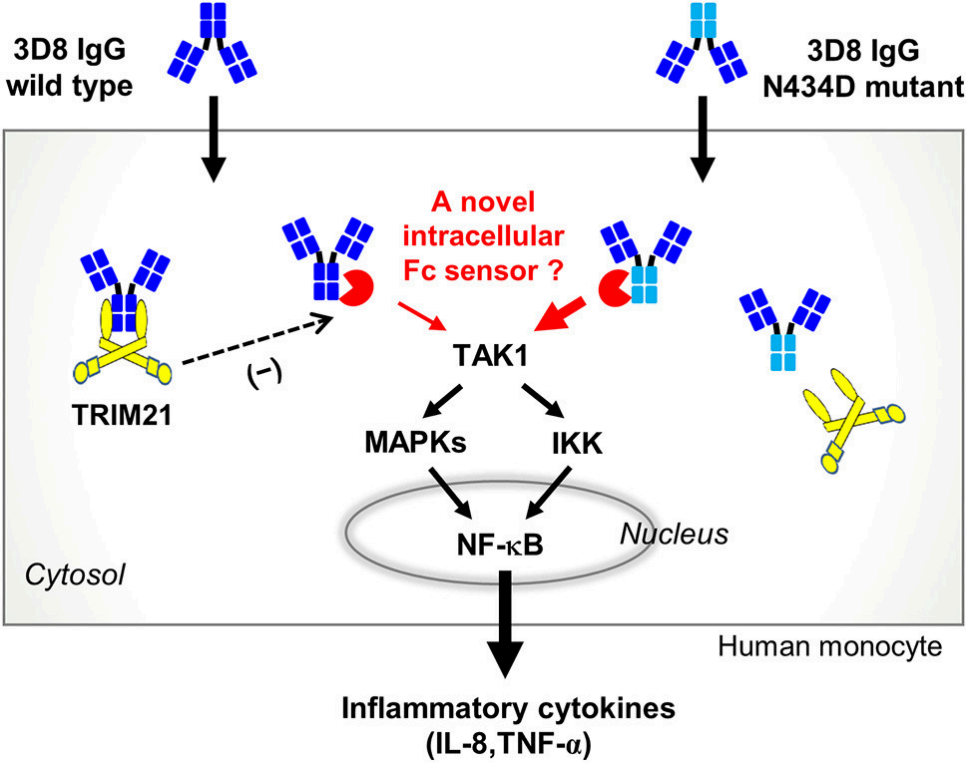
Proposed cytokine signaling pathway triggered by internalized free IgG antibody. The 3D8 IgG-N434D mutant, which is not recognized by TRIM21, induces human monocytes to produce more IL-8 and TNF-α than cells exposed to wt 3D8 IgG. An as-yet-unknown cytosolic Fc sensor may be responsible for cytokine signaling triggered by entry of free IgG to the cytosol.

Here, we identified a novel Fc-mediated cytokine signaling pathway in human monocytes stimulated by an internalizing IgG; this pathway is distinct from that mediated by TRIM21, a cytosolic Fc receptor. By interacting with internalized IgG, TRIM21 seems to act as a negative regulator of an as-yet-unknown Fc-mediated cytokine signaling pathway (Figure 5). Such a pathway might employ a novel cytosolic Fc sensor (a proposed pathway is summarized in Figure 7). In terms of the functions of TRIM21, our finding that it is not a key molecule that mediates pro-inflammatory cytokine production by internalized IgG is fairly surprising.

Upon infection of diverse cell types, TRIM21 triggers intracellular degradation of pathogens and innate immune signaling. Antibodies bound to invading pathogens are carried into the cell cytosol where they are recognized by TRIM21, which then promotes degradation of infected pathogens by recruiting the ubiquitin-proteasome system (1, 18). Simultaneously, TRIM21 potentiates innate immune responses by activating NF-κB, AP-1, and IRF transcription factors, which regulate cytokine transcription (47, 48). Recent studies examined TRIM21-mediated protein degradation mechanisms with a view to developing strategies for rapid removal of endogenous target protein antigens; strategies included delivering IgG antibodies directly to the cell cytosol or inducing expression of genes encoding IgG molecules that recognize endogenous target proteins in the cytosol (48, 49).

TRIM21-mediated immune signaling mechanisms play a role in infection by invading pathogens and in internalization of large, insoluble, antibody-bound antigens (even inanimate objects such as antibody-coated latex beads); indeed, transfection of antibody-coated latex beads causes TRIM21-induced activation of NF-κB, whereas transfection of antibodies or beads alone does not (2, 4, 50). Recently, however, internalization of a cell-penetrating IgG1 antibody targeting the HBx protein of hepatitis B virus stimulated TRIM21-mediated degradation of HBx and simultaneously activated NF-κB and AP-1 (48). In this context, it is plausible that internalizable 3D8 IgG antibodies cross-react with mRNAs in the cytosol to form ICs, which may trigger TRIM21-mediated cytokine signaling via NF-κB. However, 3D8 IgG-mediated production of IL-8 and TNF-α by human monocytes was not dependent on TRIM21-mediated immune signaling mechanisms. The different cytokine signaling mechanisms activated by internalization of IgG antibodies will be the subject of further studies.

In human B cells, TRIM21 interacts with unfolded IgG1 (which is retro-transferred from the endoplasmic reticulum to the cytosol) to regulate IgG quality via the endoplasmic reticulum-associated degradation system. This is accompanied by polyubiquitination of the IgG1 heavy chain by the E3 ligase activity of TRIM21, which leads to proteasomal degradation of unfolded IgG1 (51). In the experimental setting of TRIM21-mediated rapid degradation of proteins, degradation of an intracellular target protein was accompanied by degradation of the heavy chain of an IgG antibody delivered to the cytosol (49). By contrast, we found here that neither the amount of intracellular IgG heavy chain nor its molecular mass changed, even at 4 h post-treatment of THP-1 cells (Figure 5G). This suggests that internalized 3D8 IgG molecules are not degraded by the E3 ligase activity of TRIM21. Thus, the role of 3D8 IgG is independent of TRIM21.

Some studies focused on delivery of antibodies specific for intracellular targets (52). The presence of free IgG antibodies in the cytosol is unusual, and in the absence of infection it may be recognized as a danger signal by the host cell. Our findings suggest that free IgG antibodies may reach the cytosol of human monocytes (even antibodies that are not specific for DNA), where they induce production of pro-inflammatory cytokines in an Fc-dependent manner. Therefore, adverse cytokine responses triggered by delivery of exogenous IgG antibodies may need to be considered when attempting to modulate the function of cytosolic target molecules.

## Author contributions

HP designed and performed the research. MK, YS, and M-YC analyzed the data. YH performed the experiments. M-HK wrote the manuscript.

### Conflict of interest statement

The authors declare that the research was conducted in the absence of any commercial or financial relationships that could be construed as a potential conflict of interest.
